# Ectodermal Dysplasia with Amastia: A Case of One-Step Reconstruction

**DOI:** 10.1155/2009/927354

**Published:** 2009-05-04

**Authors:** M. Klinger, F. Caviggioli, B. Banzatti, C. Fossati, F. Villani

**Affiliations:** Unità Operativa di Chirurgia Plastica 2, Cattedra di Chirurgia Plastica, Istituto Clinico Humanitas (IRCCS), Università degli Studi di Milano, Via Manzoni 56, 20089 Rozzano, Italy

## Abstract

*Background*. Female patients presenting amastia associated with ectodermal dysplasia are not frequently encountered, but they are of great clinical interest and surgically demanding. Traditionally, skin alterations related to Ectodermal Dysplasia have addressed plastic surgeons to perform a two-stage approach in amastia associated with this congenital pathologic condition. This article describes an alternative method for correcting this deformity trough a mammary reconstruction in one surgical stage. *Materials and Methods*. We report a case of 26-year-old female patient with bilateral amastia associated with ectodermal dysplasia. Amastia in this patient was treated with implantation of subpectoral silicone gel prostheses, without previously breast tissue expansion. *Results*. At 18 months of follow-up after surgey, there were no complications and excellent cosmetic results were achieved. Patient and surgeon satisfaction was high and the patient underwent a bilateral areola-tattoo. *Conclusions*. One-stage mammary reconstruction have showed to be a reliable and effective technique also when amastia is associated with Ectodermal Dysplasia, suggesting a still satisfying biomechanical performance of the skin in this pathology.

## 1. Introduction

Ectodermal dysplasias (EDs) are a group of pathological conditions characterized by congenital defects that involve ectodermal structures and their appendages (hair, nails, teeth, and sweat glands) [1]. EDs are rare syndromes and their incidence is estimated in about 7 cases in 10 000 births [1]. More than 170 different clinical conditions have been described as ectodermal dysplasias, with extremely varied manifestations and a large superimposition of clinical features, some of them observed more frequently (Table 1) [2–4].

Amastia is a rare congenital deformity, characterized by uni- or bilateral absence of breast and nipple-areola complex. In 1965 Trier esteemed 43 cases described in a period of 126 years, with an incidence of 1 case every 3 years [5]. While this condition can be associated with ectodermal dysplasia, when we conducted a thorough PubMed literature search we were able to retrieve only two cases of bilateral complete absence of breasts and ectodermal dysplasia in female patients [6, 7]. A wide range of surgical techniques have been used to correct congenital malformation of the breast including one-stage reconstruction, use of expander implant permitting gradual augmentation of small breast and flaps [8, 9], but the concurrent presence of ectodermal structures dysplasia makes breast reconstruction particularly challenging. Two surgical stages approach has been used to correct amastia in female patients with ectodermal dysplasia in cases reported so far [6, 7].

We present a mammary reconstruction with breast implants in one surgical stage in a patient with ectodermal dysplasia associated with a complete amastia with the presence of a hint of bilateral nipples.

## 2. Case Report

**Figure 1:**
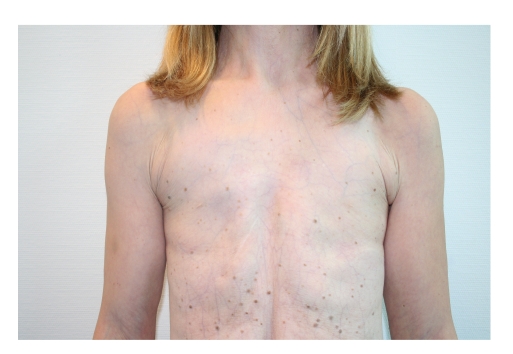
Preoperative frontal view, showing complete amastia with the presence of a hint of bilateral nipples.

**Figure 2:**
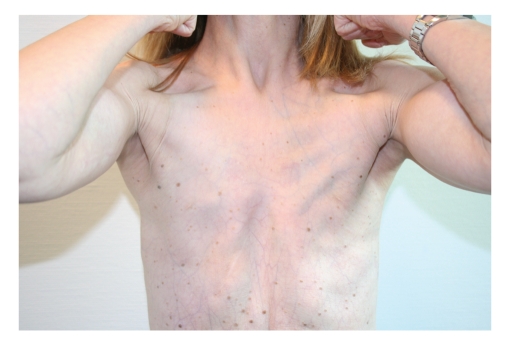
Preoperative view. Note thin skin with visible superficial vascular weave and hypofunction of pectoralis muscle.

**Figure 3:**
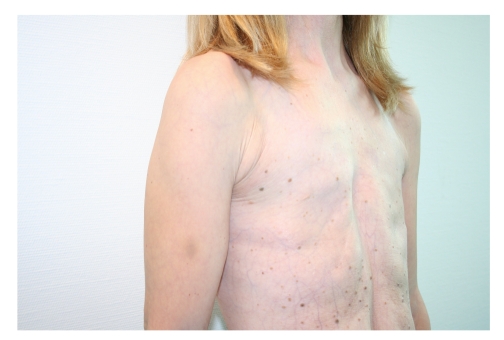
Preoperative oblique view.

**Figure 4:**
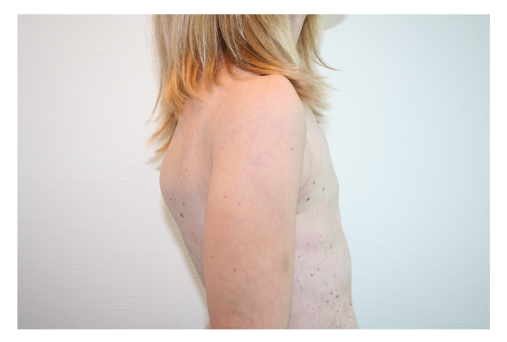
Preoperative lateral view, showing total absence of the mammary mound.

In June 2006, a 26-year-old woman was referred to us requesting correction of bilateral amastia (see Figures 1, 2, 3, and 4).

Our patient presented amastia associated with pectus excavatum and ectodermal alterations that included: complete alopecia, dystrophic nails, and convergent strabismus. An abnormal adipous tissue distribution with an increased deposition focused on superior arms and sacrolumbar region and hypotrophy of Bichat's fat pad (Corpus adiposum buccae) were also present. Patient's skin appeared thin with visible superficial vascular weave all over the body. The linea alba, a fibrous structure composed mostly of collagen connective tissue, was strictly adherent to the upper layers of the skin, without fat interposition.

**Figure 5:**
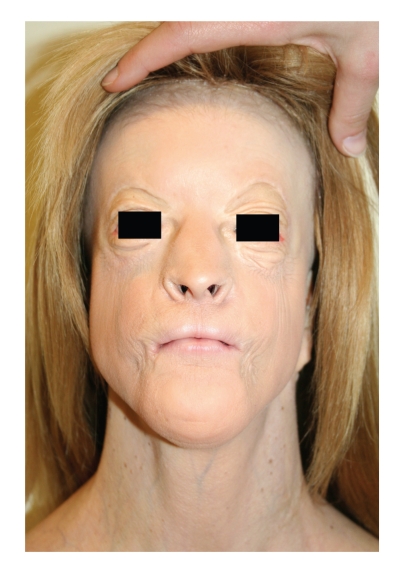
Facial ectodermal alteration.

At birth she also suffered from hypodeveloped auricles and lips and corneal ulcerations caused by bilateral absence of superior and inferior eyelids (see Figure 5). These alterations were treated with several reconstructive surgeries in her childhood. When she was one-month-old, two cutaneos biopsies showed hypotrophic cutaneous appendages, especially sweat glands. Karyotype analysis was normal. Clinical and histologic data allowed genetists and dermatologists to make a diagnosis of ectodermal dysplasia.

Amastia in this patient was treated with mammary reconstruction in one surgical stage, adapting standard techniques of breast augmentation to this particular case. No subpectoral tissue expansion was performed before the implantation of silicone gel prostheses.

 Preoperative tests included haematochemical parameters, electrocardiogram, and anesthesiologic visit. The patient also performed a thorax Nuclear Magnetic Resonance to evaluate chest wall and the major pectoralis muscle trophysm. Antibiotic prophylaxis was performed by 2 g of Cefazolin i.v. before surgery. Cefixime 400 mg 1 per day was administered orally for 8 days after surgery.

The procedure was performed in general anesthesia. A bilateral cutaneous periareolar-like incision was made at the level of the fifth rib. After skin incision, dissection with an electro-knife followed, stopping just above the external fascia of the pectoralis muscle. The lateral border of the pectoralis major muscle was identified and a subpectoral dissection was performed, creating bilateral partial submuscular pocket. In particular, the VI and VII costal origins were disinserted. After accurate haemostasis and the placement of bilateral drains, implants (Allergan style 410 MF 255 cc) were inserted into the previously created partial submuscular pockets. Subsequently, a new mammary mound and an inframammary fold were created. The inframammary fold resulted simply by the placement of the implant, without fixing sutures. The overlying skin was partially recruited from the surrounding regions (i.e., abdomen), due to its particular laxity and sliding properties. Wounds closure was completed in layers, using intradermic suture in monocryl 3–0 for skin closure.

After 6 months a bilateral areola-tattoo was performed with a tattoo machine.

**Figure 6:**
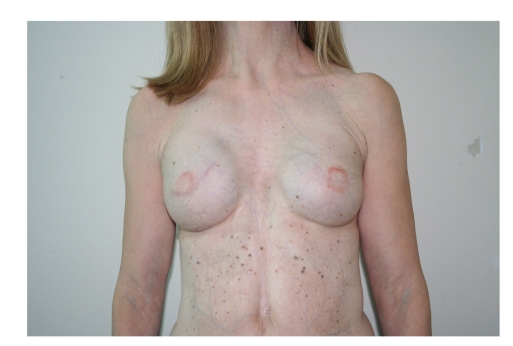
Postoperative view 18 months after one sugical stage mammary reconstruction and a bilateral areola-tattoo.

**Figure 7:**
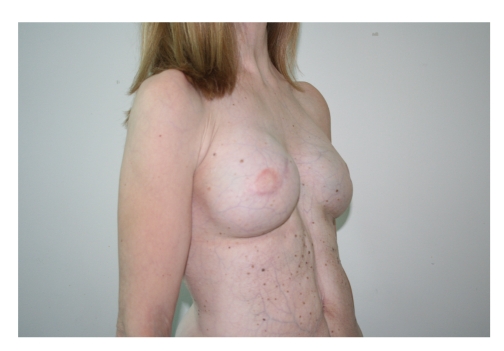
Postoperative oblique view.

**Figure 8:**
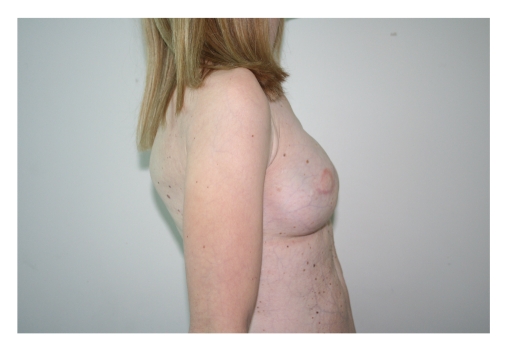
Postoperative lateral view, showing a good projection of the reconstructed breast.

Patient was followed up for 18 months and at each clinical examination the results were photographically documented (see Figures 6, 7, and 8).

## 3. Results

Surgical procedure and anaesthesia were well tolerated by the patient and there were no intraoperative or early postoperative complications. There were no evidence of bleeding or hematoma and drains were removed two days after surgery, when the patient was discharged. 

At a short-term follow-up visit there was no evidence of flogosis, infection, or wound dehiscence. No late seroma or hematoma developed thereafter. The skin covering the implants showed no sign of sufferance.

Clinical assessment done at 6 months after mammary reconstruction showed soft breast and good scarring. There was no capsular contraction (Baker Stage I). 

There were no complications over the entire post surgical surveillance period.

The patient was satisfied with the results and finally underwent a bilateral areola-tattoo.

## 4. Discussion

Mammary reconstruction in female patients with amastia and ectodermal dysplasia reported in literature has been described as two surgical stages procedures, with mammary skin expansion followed by the placement of definitive breast implants [6, 7]. 

For the correction of the bilateral amastia in this patient we took into consideration different approaches:

1. two-stage mammary reconstruction, with placement of breast tissue expanders, followed by skin expansion and the placement of definitive breast implants.
2. one-stage mammary reconstruction by breast implants;
3. mammary reconstruction by local or free flaps;

Local random advancement flaps (e.g., thoracoabdominal flap), as well as Latissimus Dorsi (LD) flap, had three disadvantages: uncertain vascularisation, excessive invasivity, and insufficient volume provided. Pedicled Transverse Rectus Abdominis Muscle (TRAM) or Deep Inferior Epigastric Perforator (DIEP) flaps were excluded also because the anatomical situation of the thin abdomen of patient did not allow harvesting all the skin in subumbilical region.

Our main concern about one-stage reconstruction by implant was the presence of a very thin skin and a possible hypotrophy of pectoralis muscle that could imply cutaneous sufferance or implant migration. We finally decided for this technique during the operation, when we noticed a normotrophic pectoralis muscle. For this reason, two-stage procedure with tissue expansion was not required.

We also planned the tattooing of the hypopigmented areolar region; for this reason infra-areolar incision could be well concealed, instead of other approaches (e.g., inframammary or axillary incision).

We conclude that the absence of significant complications and the final good cosmetically results validate our choice (one-stage mammary reconstruction) in this particular case, suggesting a still satisfying biomechanical performance of the skin also in patients with ectodermal dysplasia.

## 5. Conclusions

Mammary reconstruction in female patients with amastia and ectodermal dysplasia can be performed in one surgical stage, despite the concerns about cutaneous sufferance or implant migration. The high satisfaction both of patient and surgeon and the absence of complications suggest that this technique is reliable and effective in such patients and that the indication could be extended to other forms of amastia. However, due to the variety of aetiopathological forms and individual features of amastias, it is hard to state precise and specific indications in other similar conditions.

Although our results have been satisfactory, experience with this procedure is limited due to infrequent presentation of patients.

## Figures and Tables

**Table 1:** Manifestations of ectodermal dysplasia more frequently observed.

Structure involved	Clinical manifestations
Skin	Dry scaling skin, hypopigmentation, and wrinkles
Glands	Reduction of sweating, and salivary glands malfunction (xerostomia)
Hair	Sparse and curly hair, alopecia, and absence or malformation eyebrows
Facial changes	Dysmorphic features, facial malformations
Eyes	Corneal dysplasias, cataract, strabismus, and decreased lacrimation
Teeth	Hypodontia, anodontia, prone to caries
Nails	Leukonychia, distrophic, and malformed nails
